# Assessing Green Methods for Pectin Extraction from Waste Orange Peels

**DOI:** 10.3390/molecules26061766

**Published:** 2021-03-21

**Authors:** Laura Benassi, Ivano Alessandri, Irene Vassalini

**Affiliations:** 1 B+LabNet, Environmental Sustainability Laboratory, University of Brescia, Via Branze 45, 25123 Brescia, Italy; laura.benassi@unibs.it; 2 INSTM, U. d. R. Brescia, Via Branze 38, 25123 Brescia, Italy; 3 Chemistry for Technologies Laboratory, Mechanical and Industrial Engineering Department, University of Brescia, Via Branze 38, 25123 Brescia, Italy; 4 CNR-INO, Via Branze 38, 25123 Brescia, Italy; 5 Department of Information Engineering, University of Brescia, Via Branze 38, 25123 Brescia, Italy

**Keywords:** sustainability, pectin, hydrogels, green extraction, orange peel, food waste, energy consumption

## Abstract

In this work, we assess three different methods for the extraction of pectin from waste orange peels, using water as extracting solvent. “Hot-water”, Rapid Solid Liquid Dynamic (RSLD) and microwave-assisted extractions have been compared and evaluated in terms of amount and quality of extracted pectin, as well as embodied energy. This analysis provides useful guidelines for pectin production from food waste according to green procedures, enabling the identification of acidic “hot-water” as the most sustainable extraction route.

## 1. Introduction

Pectins are polysaccharides contained in the cell walls of plants characterized by α-(1 → 4) glycosidic linkages [1,2]. They are structural compounds involved in plant growth, antimicrobial inductors and responsible for ion balance in plants. Under the term “pectin”, it is possible to identify a number of polymers that vary according to their molecular weights, chemical configurations, and neutral sugar contents, since different plants produce pectins with different functional properties.

Pectins have been intensively investigated in recent years from a structural point of view and, thanks to their gelling properties, they are frequently employed in the food industry. More recently, the possibility of using them as starting materials to produce hydrogels has been evaluated, especially thanks to their biocompatibility, abundance and cheapness. Hydrogels are three-dimensional crosslinked polymers swollen by water [3] that can find wide applications in the everyday life, such as in hygiene products, contact lenses and patches for wounds. More advanced technological applications can be found in the fields of biomedical and tissue engineering [4], electronics [5] and environmental remediation [6]. Despite that different artificial polymers can be employed for the production of hydrogels with high performances, problems related to their biocompatibility during their use or disposal at the end of their life can arise. These considerations have led to the development of hydrogels starting from natural biopolymers, such as cellulose (derived from wood or bacteria), alginate (derived from seaweeds), chitin/chitosan (derived from crustaceans or mushrooms) or pectin (derived from plants), which are safe and biocompatible. However, the environmental sustainability of the production process is not granted. As recently analyzed [7], the environmental sustainability of the production of chitin/chitosan-based hydrogels is limited by the employment of strong acids and bases, baneful solvents and harmful crosslinkers. Moreover, extraction and processing require the use of a huge amount of water. This fact has led to the development, in very recent years, of alternative “green” strategies both for chitin/chitosan extraction and hydrogel formation. Pectin is affected by analogous issues—it should be extracted from natural sources (better if discarded by-products of other process) using green solvents and consuming low amounts of energy. Further production of pectin-based hydrogels should follow the same environmentally friendly criteria.

Pectin can be obtained from fruits and vegetables scraps—mainly citrus or apple peel [8]. In the case of pectin extracted from citrus fruits, in particular from oranges, high reliability and economic value are the main attractions of processing these by-products. In fact, it is estimated that almost 60% of the orange weight is treated as waste [9] and pectin and other valuable compounds, such as organic acids [10], are included in this percentage.

According to the 2030 Agenda for Sustainable Development adopted by United Nations, the recovery of substances from something that is already considered a waste becomes useful and advantageous [11] and is in line with the principle of green chemistry and circular economy.

In this view, different techniques are available for pectin extraction from food waste. However, most of the conventional routes are based on acidic hot extraction and employ chemicals with high environmental impacts, such as hydrochloric acid, nitric acid or sulfuric acid [12]. In addition, they require heating (80–100 °C), long extraction times (30–180 min) and they lead to low extraction yields (2.5–30%). Volatilization of compounds or degradation of high-quality substances may occur [12]. Recently, green extraction protocols have been proposed, which are promising options for making pectin extraction more sustainable, especially thanks to the reduction in operation time and limitation of high-impact chemicals [13]. These are based on the use of Deep Eutectic Solvents (DESs), thanks to their insignificant volatility at room temperature and their capability to form homogenous solutions with water [14], organic acid [12] and/or pure water as extracting solvents [15]. Additionally, innovative extraction choices are based on the exploitation of ultrasound or microwave-assisted technique, due to their low energy and reagent consumption, shorter treatment time and greater safety of the operators, in comparison to the conventional extraction techniques [9,12]. However, an efficient, reliable, economic, reproducible and environmentally safe extraction method is still sought after [16].

In particular, a clear indication of the most sustainable and, in parallel, efficient green method to extract hydrogel-grade pectin from food waste is missing.

In this paper, we examine different pectin extraction methods based on the use of water as extracting solvent: “hot-water” (with or without acid assistance), rapid solid liquid dynamic (with or without acid assistance) and microwave extractions. These are compared in terms of extraction yield, quality of obtained pectin (especially, esterification degree), use of additional reagents and embodied energy. Although no statistical analysis could be carried out due to the limited number of experiments, this study will allow for the identification of optimal solutions in view of a fully sustainable production of pectin, providing a guideline for the design of extraction protocols.

## 2. Materials and Methods

### 2.1. Orange Peel

Orange peels (OPs) were collected from domestic waste of households in Brescia, Italy. The fresh peel was sorted and thoroughly washed to remove wax or other residues. Fresh orange peels were processed in different way: cut into coarse pieces (size > 5 mm), ground (size < 2 mm) or freeze-dried (pieces size < 5 mm). Fresh coarse orange peels were subjected to pectin extraction after being washed and cut. Ground fresh orange peels were obtained by 6 s treatment of a Waring MX1200XTX X-Prep and dried under aspirating hood (Asalair Carbo, Cernusco sul Naviglio (Mi), Italy) for 10 h. Freeze-drying (Edwards Modulyo EF4 1044, Irvine, CA, USA) was performed overnight on fresh coarse pieces of orange peels.

### 2.2. Common Pretreatment

All OPs were subjected to drying until no variation of weight was observed. Dried samples were stored in bags in a dry environment before experimental analyses.

Microwave (MW) pretreatment was applied in extractions 1, 2, 3, 5 and 8. It was performed by placing OP in a microwave oven (Olimpic 52603) and heating at 550 W for 5 min.

### 2.3. “Hot Water” Method

Extraction 1: Fresh orange peels were microwave pretreated for 5 min at 550 W. Then, OPs were mixed with milli-Q water with liquid/solid ratio (LSR) equal to 20. The beaker was covered, and the mixture was kept at 70 °C under continuous stirring for 60 min.

Extraction 2: Fresh OPs were microwave pretreated for 5 min at 550 W. Then, OPs were mixed with milli-Q water with LSR equal to 40. The beaker was covered, and the mixture was kept at 90 °C under continuous stirring for 180 min.

Extraction 3: Fresh OPs were grinded, air-dried and microwave pretreated for 5 min at 550 W. Then, OPs were mixed with milli-Q water with LSR equal to 20. The beaker was covered, and the mixture was kept at 70 °C under continuous stirring for 60 min.

Extraction 4: Ground OPs were added to an acid solution composed of milli-Q water and commercial citric acid at pH = 1.5, with LSR = 20. The beaker was covered, and the mixture was kept at 70 °C under continuous stirring for 60 min.

### 2.4. Rapid Solid Liquid Dynamic (RSLD) Extraction

The RSLD extraction is based on a suction effect, involving a compression of extracting solvent on solids at a pressure of about 8–9 bar for a fixed time, and followed by an immediate decompression at the atmospheric pressure. Both a rapid release of the extracting liquid and mechanical pressure gradient transport of the extractable compounds from the inside of a solid matrix towards the outside occurre Water was employed as the extracting liquid and the material underwent a treatment composed of cycles of static/dynamic phase, during which a piston pressed the material until 8 bar for 10 s and released. This static/dynamic phase (piston hit) was repeated 12 times for each cycle.

Extraction 5: Fresh OPs were microwave pretreated for 5 min at 550 W. Then, OPs were inserted into a porous bag and placed in the extractor chamber of the instrument (Naviglio^®^, Napoli, Italy). Milli-Q water was added into the chamber with LSR of 4. The extractive phase lasted for 5 h.

Extraction 6: Ground OPs were inserted into a porous bag and placed in the extractor chamber of the instrument (Naviglio^®^). Milli-Q water was added into the extractor chamber with LSR of 10. The extractive phase lasted for 3 h.

Extraction 7: Ground OPs were inserted into a porous bag and placed into the extractor chamber of the instrument (Naviglio^®^). Milli-Q water was added into the extractor chamber with LSR of 10. The extractive phase lasted for 5 h.

Extraction 8: Freeze-dried OPs were microwave pretreated for 5 min at 550 W and inserted into a porous bag and placed into the extractor chamber of the instrument (Naviglio^®^). Then, an acid solution composed of milli-Q water and citric acid (pH = 1.5) was added with LSR equal to 10. The extractive phase lasted for 5 h.

### 2.5. Microwave-Assisted Extraction

Extraction 9: Freeze-dried OPs were introduced into the reaction vessel of the CEM Discover microwave oven (Matthews, NC, USA) with a microwave safe stirrer bar and milli-Q water, with an LSR equal to 6. The instrument parameters were set as: maximum temperature—110 °C, ramp time—5 min, heating time—5 min, maximum power—300 W, medium stirring.

Extraction 10: Ground OPs were introduced into the reaction vessel of the CEM Discovery microwave oven with a microwave safe stirrer bar and milli-Q water, with an LSR equal to 6. The instrument parameters were set as: maximum temperature—110 °C, ramp time—5 min, heating time—5 min, maximum power—300 W, medium stirring.

### 2.6. Common Post-Treatment: Pectin Isolation and Drying

After the extraction phase, OPs were removed by filtration from the extracting solution. Pectin precipitation was achieved with the addition to the liquid of 98% ethanol, with an ethanol:solution ratio of 1:1, except for microwave-assisted extractions where the ethanol:solution ratio was set to 1.5:1. The solution is then left to ripen for at least 20 h.

Precipitated pectin was recovered by centrifugation (OHAUS Frontier 570, Nanikon, Switzerland) for 15 min at 4000 rpm (acceleration value of 9, deceleration value of 3 relative centrifugal force (RCF)). Isolated pectin pellets were washed twice with 98% ethanol (total 10 mL). The extracted pectin was air dried.

### 2.7. Characterization

#### 2.7.1. FT-IR

Fourier Transform Infrared (FTIR) Spectroscopy was used to verify the chemical nature of the extracted material.

Each sample was incorporated with KBr (weight ratio KBr:pectin 10:1) and pressed into pellets. The FTIR spectra were collected at the absorbance mode in the region of 500–4000 cm^−1^ with an FTIR Vertex 70v Bruker instrument (Billerica, MA, USA). Every measure is the averaged result of 128 consecutive acquisitions.

Commercial pectin (Sigma-Aldrich P9135, St. Loius, MO, USA) was analyzed for comparison.

#### 2.7.2. SEM

The morphology of extracted pectin was investigated through scanning electron microscopy, Zeiss LEO EVO 40 (Oberkochen, Germany). Different magnification images (5000×, 2500×, 500× and 66×) were recorded. Commercial pectin (Sigma-Aldrich P9135) was analyzed for comparison.

#### 2.7.3. Thermogravimetry-Differential Thermal Analysis (TG-DTA)

Thermal properties of the extracted samples of pectin were studied by means of thermogravimetric analysis (TGA), performed with a TA Instrument SDT Q600 (New Castle, DE, USA) apparatus equipped with Universal Analysis 2000 software. In argon atmosphere, the samples were placed in an alumina pan and heated up to 650 °C with a scanning rate of 10 °C/min.

#### 2.7.4. Determination of Degree of Esterification

The degree of esterification (DE) of extracted pectin was calculated by means of the titration method [17] with slight modifications, as described by Hosseini et al. [18]. Dried pectin (0.1 g) was wetted with 2 mL ethanol. Distilled water at 40 °C (20 mL) was added and kept under stirring. After complete dissolution of the sample, 5 drops of phenolphthalein were added, and the solution was titrated with 0.1 M NaOH (V_1_). Then, 10 mL of 0.5 M NaOH was added. The sample was left to stand for 20 min for hydrolysis. Following, 10 mL of 0.5 HCl was added under stirring till the pink color disappeared. At the end, 5 drops of phenolphthalein were added, and the titration occurred through the addition of 0.1 M NaOH until a slight pink color persisted (V_2_). DE of the pectin was calculated according the following equation (Equation (1)):

(1) $$\%\;\mathrm{DE}=\frac{V_{2}}{V_{1}+V_{2}}\times 100$$

Commercial pectin (Sigma-Aldrich P9135) was analyzed for comparison.

#### 2.7.5. Calculation of Energy Demand

The estimation of total energy demand for each extraction protocol was calculated on the basis of instrumental energy consumption and chemical embodied energy.

The values of the energy consumed by each instrument involved in each extraction protocol have been calculated by multiplying its power for the operation time needed to produce 10 g of pectin.

The values of embodied energy linked to additional chemicals have been calculated on the basis of the amount of ethanol employed for the precipitation of extracted pectin and the value of its embodied energy (MJ/Kg) acquired from the openLCA software (Green Delta, version 1.10.3, Berlin, Germany, 2006) (considering ethanol produced by means of ethylene hydration). In the case of extractions 4 and 8, the contribution of citric acid’s embodied energy acquired from the openLCA software (considering production through a fermentation process) was added by multiplying it by the amount of citric acid needed for the production of 10 g of pectin.

Even if all these calculations are based on a certain grade of approximation, they can be used to make a preliminary comparison about the environmental sustainability of the analyzed extraction protocols.

## 3. Results and Discussion

**Figure 1:**
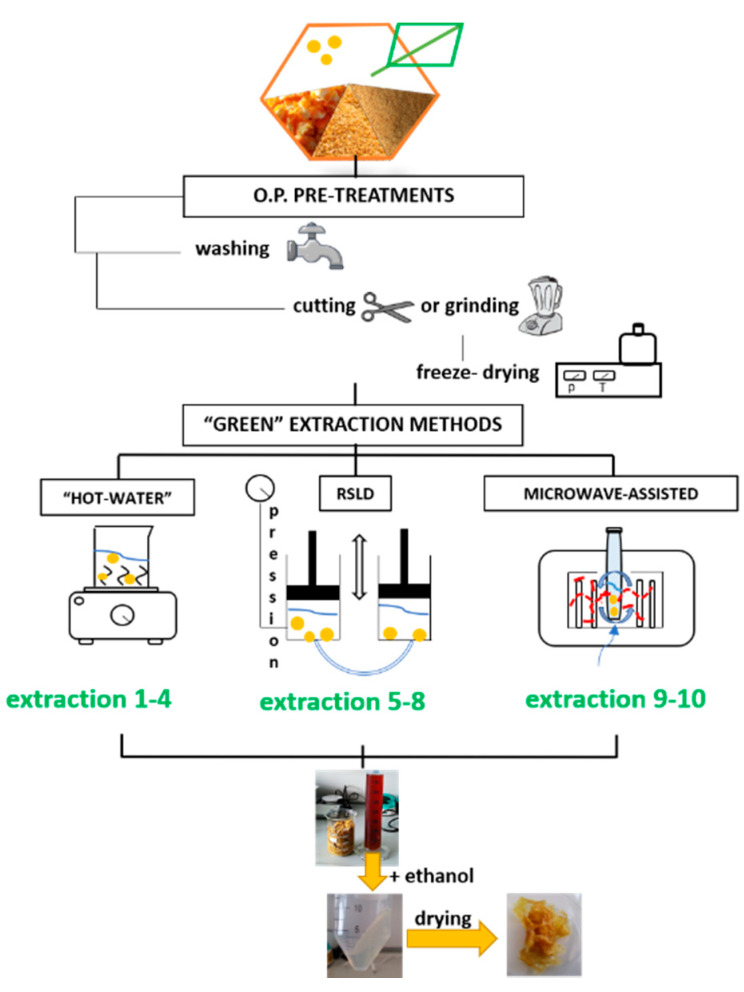
Scheme of the extraction routes investigated in this study: “hot-water”, rapid solid liquid dynamic extraction and microwave digester.

In order to identify the extraction method that enables to obtain good-quality pectin with the lowest environmental impact, three different “green” routes were evaluated: “hot-water”, rapid solid liquid dynamic and microwave-assisted extractions (see scheme in Figure 1).

The extraction procedures have been compared in terms of energy consumption, volume of ethanol used for pectin precipitation, yield and quality of extracted pectin. As the first comparative parameter, the energy consumption due to all the laboratory instruments involved in the extraction protocol (mechanical grounding, freeze-drying, heating, etc.) has been considered. For this calculation, the operation time needed for the extraction of 10 g of pectin has been taken into account. For example, in the case of microwave-assisted extraction, the sample holder enables to host only 2 g of OPs at a time, which leads to the production of 0.05–0.124 g of extracted pectin. This means that each extraction cycle should be repeated from ~80 (in the case of the highest extraction yield) to ~200 (in the case of the lowest extraction yield) times for extracting the desired amount (10 g) of pectin.

**Figure 2:**
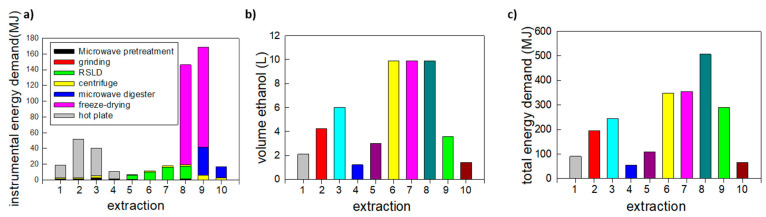
(**a**) Contribution of the different experimental steps to the energy consumed by instruments for producing 10 g of pectin following different extraction protocols; (**b**) volume of ethanol used for the precipitation of 10 g of pectin following different extraction protocols; (**c**) total energy consumed (instruments + chemicals embodied energy) to produce 10 g of pectin following different extraction protocols.

This approximate evaluation of energy investment in the extraction procedures (summarized in Figure 2a) is sufficient to draw interesting guidelines. A detailed description of the calculation of the data reported in this figure can be found in SI 1 (Tables S1–S11).

Among the different steps, freeze-drying pretreatment is the most energy demanding (126.720 MJ).

Microwave pretreatment (550 W for 5 min) consumes 165 kJ at a time, and from the literature, it is expected to conditionate the amount, quality and the esterification degree (DE) of the final pectin [19]. In fact, OP is made of polar molecules able to interact with the microwave electromagnetic field, producing heating and exerting high pressure on plant structure, with new capillary and pore formation [19]. Additionally, MW treatment inactivates the pectinmethylesterase [19], responsible of the regulation of number and distribution of free carboxyl groups along the pectin molecule [20], with a relevant increase in DE of the final product.

As shown in Table 1, freeze-drying does not enable the improvement of the extraction yields, either in the case of RSLD (extraction 8) or microwave extraction (extraction 9), so it should be avoided because of its high energy demand. Grinding entails small energy consumption (approximately 36–135 kJ, according to the amount of orange peel needed for the extraction of 10 g of pectin) and it is an important step to achieve a more uniform starting feedstock, as well as to save space for storage and optimize transport. As visible from the comparison between extractions 1 and 3 for the “hot water” method, or extractions 5 and 7 for RSLD, it does not enhance extraction yield.

Considering the energy consumed only by laboratory instruments, it is possible to make a first estimation: extraction 5 (RSLD on fresh pieces of orange peel with LSR = 4 for 300 min) is the most convenient, followed by extraction 4 (acidic “hot-water” for 5 h) and extraction 6 (RSLD on ground pieces of orange peel with LSR = 10 for 180 min).

These data have been complemented with the estimation of the amount of ethanol used for the production of 10 g pectin following different extraction procedures (Figure 2b). The volume of ethanol used for pectin precipitation is directly linked to the amount of water used during extraction, so it is mainly linked to LSR and extraction yield. As a result, extraction protocols characterized by low LSR values and high yields enable the reduction ethanol consumption. In this regard, protocols that seem more advantageous are acidic “hot-water” extraction (extraction 4), RSLD with low LSR (extraction 5) and microwave extraction on ground pieces (extraction 10).

Then, we have tried to estimate the embodied energy of the chemicals involved in the different extraction protocols: ethanol and citric acid. As visible in Figure 2b, ethanol is used in huge amount during the different protocols (ranging from 1.2 to 9.9 L for the production of 10 g of pectin), so its embodied energy (43.1 MJ/kg according to openLCA software) significantly affects the total amount of energy demand (Figure 2c). On the contrary, citric acid (embodied energy: 85.66 MJ/kg according to openLCA software) is used only in reduced amounts in extractions 4 and 8, so its contribution to the final count is limited. Details on the calculation of the contribution of chemical embodied energy can be found in Table S12. From Figure 2c, it is possible to obtain a good estimation of the total energy demand of the different extractions and it is evident that extraction 4 based on the acidic “hot-water method” is more convenient, followed by extraction 10 (microwave treatment on ground orange peel) and extraction 1 (“hot-water” on fresh pieces working for 60 min at 70 °C with LSR = 20). It is interesting to note that all the extractions based on the RSLD methods are characterized by low values of energy consumed by instruments, but the huge amount of ethanol necessary for pectin precipitation reduces their sustainability.

As regards the quality of the extracted pectin, the morphology has been evaluated by means of SEM analysis, purity has been evaluated by means of FTIR and thermogravimetric analysis, while esterification degree has been evaluated by means of chemical titration. The results obtained for all the extracted samples have been compared with those of a standard commercial sample, and they are summarized in Table 1.

**Figure 3:**
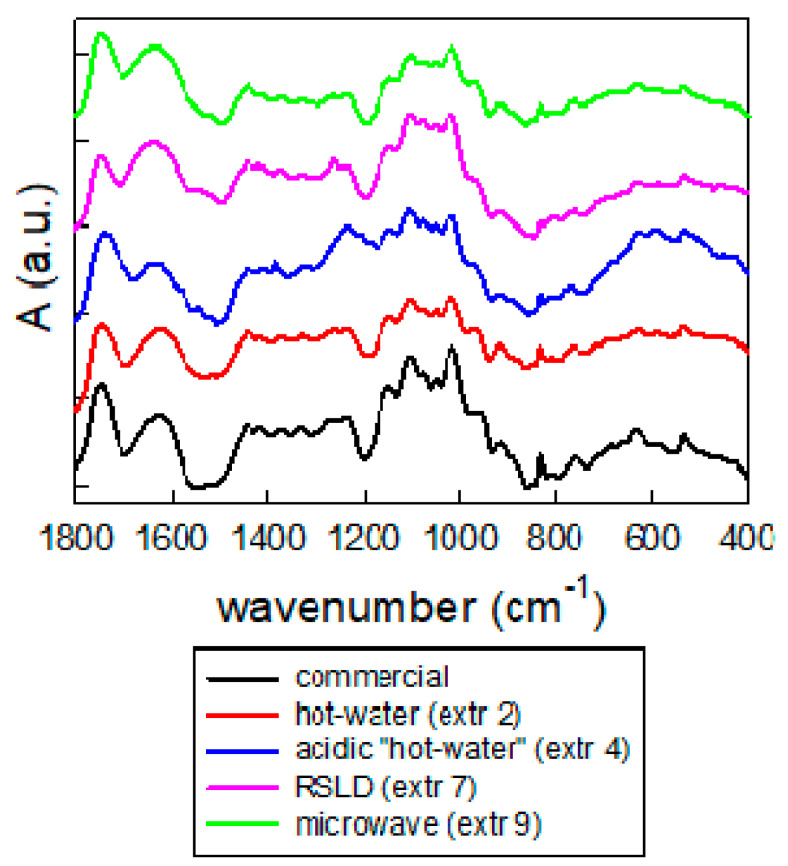
Fourier Transform Infrared (FTIR) spectra of standard commercial pectin; “hot water” extracted pectin in the absence (extraction 2) of and in the presence of citric acid (extraction 4); Rapid Solid Liquid Dynamic (RSLD) extracted pectin (extraction 7) and microwave extracted pectin (extraction 9).

The chemical nature of the extracted samples was confirmed through the recording of FTIR spectra and their comparison with the spectrum of a commercial pectin sample. In Figure 3, some representative spectra of different extraction protocols are reported, and all of them are characterized by the presence of the same main peaks: ~1740 (ν C = O in pectin methylesters), ~1620 (ν as CO_2_), 1440 cm^−1^ (νs CO_2_), 1150 (ν as O-C-O ring), 1100 (v (C-O)(C-C)), and 1010 cm^−1^ (ν(C-O),δ(C-OH)).

**Figure 4:**
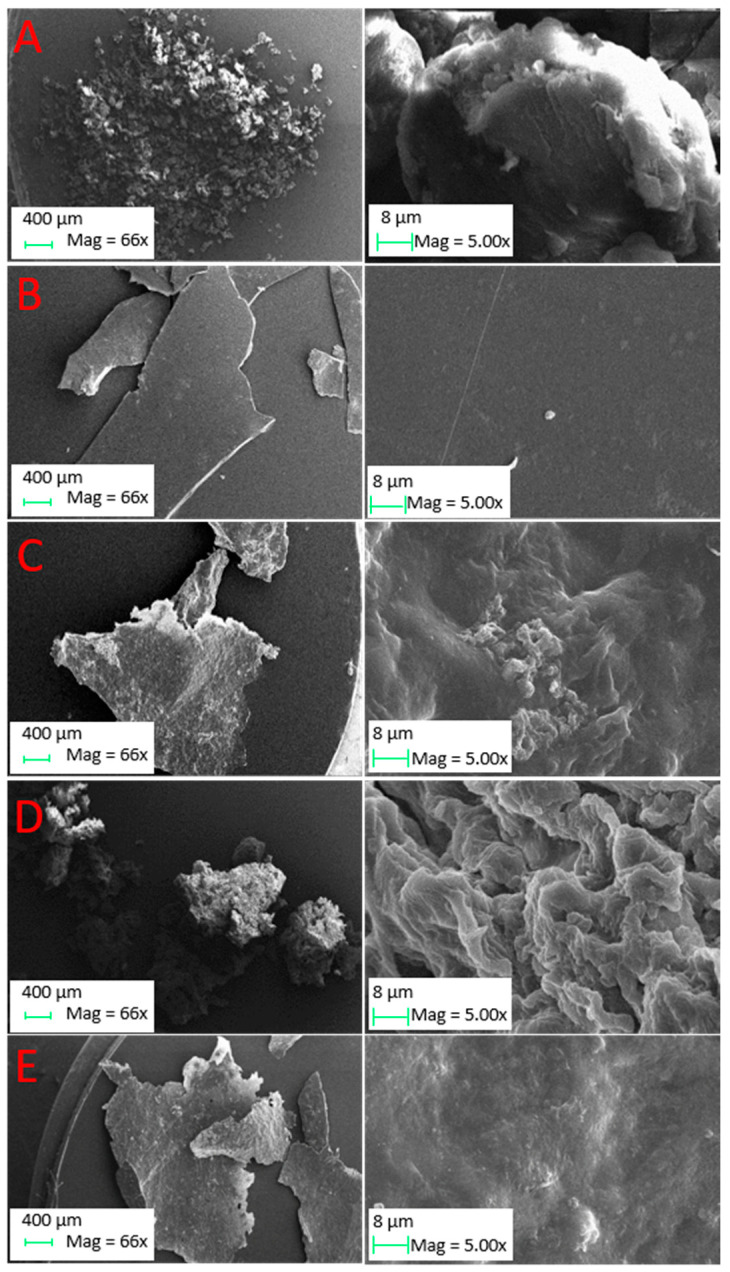
Low- and high-magnification SEM images for (**A**) standard commercial pectin; (**B**) “hot-water” extracted pectin in the absence (extraction 2) of and (**C**) in the presence of citric acid (extraction 4); (**D**) RSLD extracted pectin (extraction 7); (**E**) MW extracted pectin (extraction 9).

As regards morphology, low- and high-magnification SEM images reveal significant differences between pectins obtained through different extraction protocols. Commercial pectin (Figure 4A) exhibits a granular shape, whereas pectin obtained through the “hot-water” extraction (extraction 2; Figure 4B) shows a very flat surface. In the Supporting Information (S2) it is possible to observe the morphology of pectin obtained through “hot-water” extractions in milder conditions (extractions 1 and 3), and it is possible to notice that the final morphology is less regular. In general, we can say that considering “hot-water” extractions, the final pectin morphology is strongly influenced by different extraction parameters; in particular, by increasing the LSR ratio, extraction time and temperature, we observed a progressive surface flattening. Pectins obtained through acid-assisted “hot-water” (Figure 4C) and RSLD extractions (Figure 4D), instead, show the presence of deep cavities and stressed surface, yet to different extents in the two samples. An intermediate situation was obtained in the case of microwave-assisted extraction (Figure 4E), leading to a regular surface, with the presence of only small cavities.

Thermogravimetric analysis revealed that the curve of pectin shows three different regions (50–190 °C, 190–400 °C and 400–650 °C [21,22]). Evaporation of water is responsible for the weight loss in the first region, while the main mass loss (approximately 40%) is located in the second region, where polysaccharide decomposition occurs [23]. The third region displays the decomposition of the char [22], which is responsible of the final slow weight loss.

**Figure 5:**
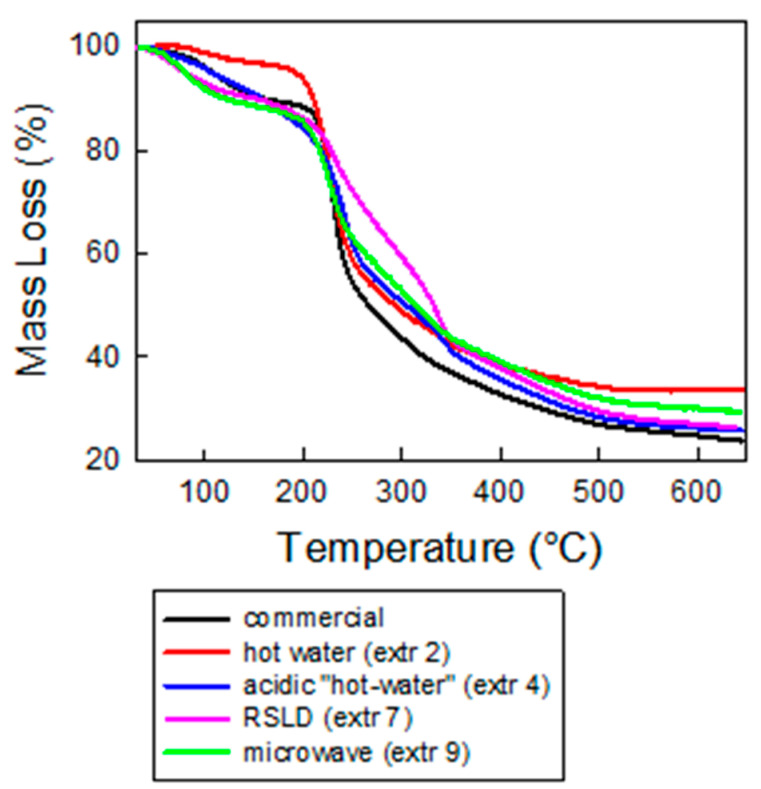
Thermogravimetric analysis (TGA) curves of pectin obtained through “hot-water” extraction in absence (extraction 2) of and in the presence of citric acid (Extraction 4), RSLD extraction (Extraction 7), microwave-assisted extraction (extraction 9) and commercial pectin.

In Table 1, the main quantitative parameters extracted from the thermogravimetric curves are reported: the temperature at which the 50% of the mass loss occurs (T_%50_), the temperature that corresponds to the maximum decomposition rate (DTG_max_), and the total mass loss at 650 °C [23]. From the obtained experimental curves (Figure 5) and the extracted data, it is possible to determine the thermal stability of the obtained samples and infer information about their purity, thanks to a comparison with commercial pure sample. In particular, it is possible to observe that the “hot-water” and microwave-assisted protocols give DTGA_max_ values that are very close to that of standard commercial pectin (228 and 227 °C vs. 231 °C), suggesting a similar thermal stability. Citric acid treatment does not drastically impact the overall quality of pectin (DTGA_max_ = 243 °C). On the contrary, the DTG curve of RSLD extraction is significantly different. In particular, we note that the DTGA_max_ value is significantly higher and that in the TGA curve there is a bump at 337 °C. According to the literature [24], this feature can be ascribed to impurities of sugars still linked to pectin or remaining starch with low molecular weight. The presence of impurities in all the extracted samples, even if in different grades, is also confirmed by the higher values of T_50%_, in comparison to the standard commercial sample, as well as the lower values of mass loss at 650 °C. This information is relevant in view of hydrogel fabrication, as these impurities may affect the time occurring to gel and the color of the final product. In addition, pectin with high thermal stability (high values of DTGA_max_, low value of mass loss@650 °C) is preferred in the food industry due to the suitability of the use as an additive in high-temperature treated food products.

In view of producing hydrogels, a fundamental parameter for the classification of pectin is the degree of methyl esterification (DE) [25]. According to this, pectin could be characterized by a high degree of methoxylation when DE is higher than 50%, or a low degree of methoxylation when DE is less than 50%. The two types of pectin are called High Methoxyl (HM) and Low Methoxyl (LM) pectin, respectively, and they are characterized by different physicochemical properties [15]. This directly impacts on hydrogel production: HM-type pectins are able to jellify more rapidly than the LM ones, requiring low pH [26]; LM pectin gelation, instead, requires metal cations and the process is based on the “egg-box” model [27].

The quantification of the degree of esterification of pectin samples has been obtained by means of chemical titration, as reported in the Section 2, and the results are summarized in Table 1.

The analysis highlights that the extracted pectins can be classified as HM and LM, according to the extraction method. All the samples extracted through the “hot-water” method are HM, while the majority of the pectins extracted through RSLD are LM, except for extraction 5. In particular, “hot-water” and RSLD extractions (extraction 5) enable the obtainment of a DE higher than samples obtained through microwave extractions and commercial pectin (DE = 50%). Regardless, within a set of the same extraction method, a high variability is observable.

### 3.1. Analysis and Optimization of “Hot-Water” Extraction

As regards the “*hot-water*” extraction method, a detailed investigation on the effects of different parameters, such as the liquid-to-solid ratio (LSR: 20–40), extraction time (60–180 min) and temperature (70–90 °C), use of fresh vs. ground orange peel (OP) and pH lowering (addition of citric acid), has been performed.

Extractions 1 and 2 were carried out from fresh OPs under two opposing conditions. Extraction 1 adopted the lowest values of the liquid-to-solid ratio, temperature and extraction time reported in the literature [28]—i.e., LSR = 20, temperature = 70 °C, extraction time = 60 min. On the other hand, extraction 2 was carried out under the following conditions: LSR = 40, temperature = 90 °C, extraction time = 180 min. Isolation and drying of pectin were performed according the same protocol for both the extractions, as described in the Section 2.

Despite the different extraction conditions, the yield was about 10% in both cases.

According to the literature, we should have expected higher values in the second extraction [28], because temperature, extraction time and LSR would have a direct impact on yield. The higher temperatures should be responsible for an increase in solubility of pectin; longer extraction time should allow the pectin mass exchange from solid particles into a solution and higher LSR should increase the contact area between the plant tissue and extraction liquid. However, waste-recovered, fresh OPs are affected by a large variability in their quality, which can mitigate or even strongly undermine the effects of temperature and extraction time increase. In addition, washing OPs to remove wax or other residues could modify the peel moisture content and consequently affect the LSR.

On the contrary, a variation of these extraction parameters modifies the DE of the final pectin, which is 78.9% in the case of milder conditions (extraction 1) and 60% in the case of more drastic conditions (extraction 2). The difference in the percentage of DE could be due to the increasing de-esterification of polygalacturonic chains caused by the combination of a high temperature and long extraction time [28].

Regarding the environmental impact due to the involved chemicals, the only contribution is given by the addition of ethanol for pectin precipitation (1:1, ethanol: water) and washing. Being the ethanol dosage related to the amount of the extraction solvent, it is evident that extraction 2 needs about the double amount of ethanol. Moreover, from the total energy demand (instrument consumption + chemicals embodied energy) viewpoint, we observe that extraction 2 requires two times (2.15) the energy consumed in extraction 1 for the production of 10 g of pectin. Therefore, we can consider extraction 1 as more sustainable than extraction 2.

Extraction 3 was performed by maintaining all the operational parameters equal to extraction 1, modifying the OP initial state (ground air-dried pieces instead of coarse fresh pieces). Grinding results in drastic and detrimental modification of all the most relevant parameters: yield, DE and instrument energy consumption. By introducing grinding, DE passes from 78.9 to 62.5% and the extraction yield from 10 to 3.5%. Even if grinding per se has a limited impact in terms of energy consumption (only 135 kJ), the consequent reduction in extraction yields leads to a significant increase in the number of repetitions of the experiment for producing 10 g of pectin, with related enhancement of ethanol amount and total energy demand, so that the final energy request in 2.7 times the energy of extraction 1. Not only the percentage of DE, but also the quality of pectin is significantly different, as observable from the analysis of FTIR spectra reported in Figure S2, which revealed the presence of impurities. Thanks to a comparison with the FTIR spectrum of depectinated residues recovered at the end of the extraction procedure, it is possible to conclude that these impurities are due to peel residues inside the pectin samples associated with proteins present in the cell walls. Their content in fruit and vegetable dry matter, in fact, could reach values close to 30% [29].

In order to combine the superior quality of pectin extracted through protocol 1 with the advantages provided by grinding in terms of handling and storage, we explored pH lowering as a way to improve the extraction yield, as already reported in the literature [25,28,30].

Thus, extraction 4 was carried out with the same parameters utilized in extractions 1 and 3 (LSR = 20, temperature = 70 °C, extraction time = 60 min with ground OP), with the only exception of pH, which was set at 1.5 by adding commercial citric acid to OP suspension in water before heating.

From a procedural point of view, the instrumentation employed is comparable with the previous extractions; however, the extraction yield was enhanced to 21%, which overwhelms the 10% yield achieved in extractions 1 and 2. This fact leads to a significant reduction in number of experiment repetitions, with consequent limitations of ethanol amount and energy consumption (for the production of 10 g of pectin, the total energy demand associated with extraction 4 is 0.6 of energy of extraction 1). Moreover, DE was raised to 87.9%, which is the highest value among the “hot-water” protocols. We note that acid-assisted extraction operates with ground OPs, allowing to obtain an optimal “hot-water” protocol that can be implemented in the hydrogel production chain.

### 3.2. Analysis and Optimization of RSLD Extraction

Rapid solid–liquid dynamic extraction is the second extraction route examined (extractions 5, 6, 7 and 8). This technique operates at environmental temperature and allows the extraction the substances avoiding their degradation. It is based on Naviglio’s principle, which takes advantage of a negative gradient of pressure between the internal part of the material and the liquid outside [31].

In extraction 5, 250 g of fresh OPs was treated in RSLD apparatus for 5 h, adding cold water with an LSR equal to 4. The LSR choice aimed at limiting the use of ethanol for pectin extraction and precipitation, being the alcohol dosage directly linked to extraction solvent quantity [15,25,32]. The advantageous quantity of treated material through this extraction route is evident (in the case of “hot-water” extractions the initial amount of OPs is limited to 15–20 g in a single experiment). The percentage of DE is around 80% and energy consumption for the production of 10 g of pectin limited to the laboratory instrumentation is 6453 kJ, less than all the “hot-water” extractions (0.6 of the instrumental energy of extraction 4). Despite the very low extraction yield (about 1.4%), the high quantity of starting material (OPs) that can be processed during one experiment enables the limitation of the number of repetitions, as well as the final energy demand due to instrumentations. On the contrary, the high starting amount of OP and water leads to huge amount of ethanol for pectin precipitation, which is responsible for the enhancement of the total energy demand for this extraction. In fact, also taking the ethanol embodied energy into consideration, the total energy demand is 2 times that of extraction 4.

On the basis of the literature and previous observations, the low extraction yields could be due to the low LSR value. Therefore, we increased the LSR ratio from 4 to 10 during extractions 6 and 7. In these cases, ground OPs were used instead of fresh OPs. The utilization of ground OPs and the increase in LSR ratio do not enable enhancement of the extraction yield, which is even reduced to 1% and which remains the main limitation of this technique. In addition, the increase in LSR ratio also leads to a significant lowering of DE, other than a significant increase in amount of ethanol.

In extractions 6 and 7, 110 g of orange peel was subjected to 3 and 5 h treatments, respectively, but this variation of operation time does not have significant effects either on yield or DE. At first sight, the reduction in operation time from 5 to 3 h should lead to a reduction in instrumental energy consumption (and this is true in the case of a direct comparison between extractions 6 and 7—the energy consumed by instruments in the case of the 5 h protocol is 1.6 times higher than the energy consumed in the 3 h protocol), but, when we compared these two extraction routes with extraction 5, we noticed a significant increase in energy consumption, which is directly linked to the lower amount of orange peel (due to the higher LSR ratio) that can be processed during one experiment. Higher values of LSR and lower amount of starting OPs, in fact, involve an increase in the number of repetitions for the extraction of 10 g of pectin. We also have to take into consideration the embodied energy associated with ethanol consumed during pectin precipitation, which has a significant impact on the calculation of the total energy demand. The amount of ethanol used in extractions 6 and 7 is more than triple that of extraction 5, with a significant enhancement of the value of the total energy demand. Considering all these aspects, the total amount of energy linked to extractions 6 and 7 is ~3.3 times that extraction 5 and ~6.4 times that of extraction 4 (the most convenient procedure).

Rapid solid–liquid dynamic extraction was performed also on pretreated freeze-dried OPs (extraction 8) for 5 h. On the basis of the low instrumental energy consumption of this type of extraction technique, freeze-dried OPs were chosen to optimize the space in the internal chamber of the instrument and because these kinds of starting conditions are common in the literature for pectin extraction [24]. In addition, in view of the improvement of the results obtained in the case of “hot-water” extraction after acidification, it was chosen to modify the pH until it reached 1.5 by adding citric acid. In this case, the effects of the pH variation are not visible, neither considering the extraction yield (about 1%) or the DE (about 30%). On the contrary, freeze-drying has a great impact on instrumental energy demand, and, overall, extraction 8 involves an instrumental energy consumption that is 8.1 times the instrumental energy of extraction 7 (same extraction parameters, except from freeze-drying). A problem related to low yield is that a huge amount of ethanol is still required, leading to a total energy demand that is 4.6 times that of extraction 5 and 9.2 times that of extraction 4.

### 3.3. Analysis and Optimization of Microwave-Assisted Extraction

Microwave-assisted extraction is the third extraction route examined (extractions 9 and 10). Extraction parameters are the same in both the protocols, with different starting materials—freeze-dried orange peels in the case of extraction 9 and ground peels in the case of extraction 10. Similarly, to what happens in the case of RSLD extraction, the addition of freeze-drying pretreatment is detrimental—it causes a reduction in the extraction yield (from 6.2 to 2.5), leading to an increase in the number of repetitions of experiments for the extraction of 10 g of pectin and of the ethanol amount (the volume used in extraction 9 is 2.5 times that of extraction 10). The consequence is a significant enhancement of energy consumption (total energy demand in extraction 9 is 4.5 times the energy demand of extraction 10). On the contrary, freeze-drying enables the increase in DE of the final pectin.

All this underlines the fact that, if we consider microwave-assisted extraction without freeze-drying pretreatment, it can be considered a good alternative to the “hot-water” method. In fact, the total energy demand as well as the amount of ethanol of extraction 10 is comparable (1.2 times) to that of extraction 4.

### 3.4. Comparison of Extraction Yield, DE and Energy Input

**Figure 6:**
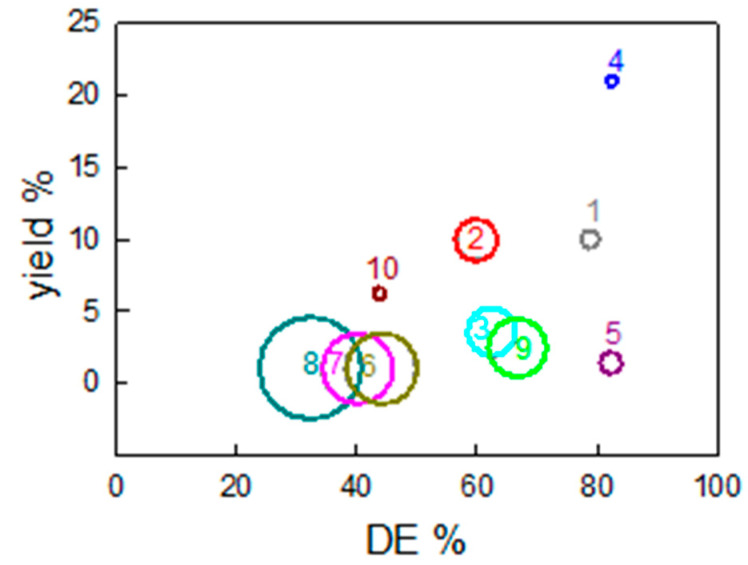
Comparison of extraction yield, esterification degree (DE) and energy investment necessary for the production of 10 g of pectin (amplitude of circle area) for each extraction. The number in/near the circle represents the extraction protocol.

These results can be compared with each other in terms of the three main parameters that are relevant in view of the final application of pectin: extraction yield (x-axis), % of DE (y-axis) and total energy demand (circle area) for the production of 10 g of pectin. Clearly, since the different extraction protocols are characterized by different extraction yields, they require variable amounts of starting OPs to produce the same amount of pectin. Figure 6 shows a synoptic comparison of all these parameters and the number in/near the circle represents the type of extraction, as indicated above.

As already observed, extraction 4 resulted in the highest values of % DE and extraction yield. This means that it requires the lowest amount of starting OP among all the tested extraction methods. As the energy input is the lowest obtained, we can conclude that acidic-assisted extraction is the optimal route to obtain pectin from orange peel waste through a simple “hot-water” approach. This consideration is further supported by the reduced amount of ethanol consumed for pectin precipitation, as visible in Figure 2b. The use of citric acid instead of a more conventional mineral acid to reduce the pH of the extraction solvent (water) would minimize the environmental impact, introducing a synergistic loop in the production cycle. In fact, citric acid is abundant in citrus fruits and it could be recovered from extraction experiments. For example, the literature reports that citric acid can be easily extracted from citrus fruit peel (including orange peel) or produced through fungi, such as A. Niger, which can be intentionally grown on the citrus fruit peel [33]. This opens an intriguing circular perspective, which can further promote the sustainability of the whole process.

Extraction 4 outperformed not only the other “hot-water” extractions, but all the considered protocols.

In particular, RSLD extractions (extractions 5–8) are characterized by low extraction yields and huge consumption of ethanol, which significantly enhance the total energy demand. Using fresh OP in this technique and considering low values for the LSR (extraction 5), enables the reduction in the amount of ethanol for pectin precipitation and obtain a high (>80%) value of DE %, which is crucial for specific applications [34,35], but the total energy demand still remains higher than acidic “hot-water” extraction. The most intriguing aspect of this extraction protocol is that, by varying operational conditions, RSLD can generate LM pectins (between 30 and 40% of DE) or HM pectins (up to 80%). Thus, this extraction method can be adapted to the hydrogel versatility. Moreover, some optimization procedures could be studied: reducing the relative amount of water and ethanol or trying to reprocess several times the high amount of pectin that remains in the extraction medium in order to maximize recovery.

Finally, microwave extractions (9 and 10) are characterized by intermediate extraction yield and DE. Without considering freeze-drying pretreatment (extraction 10), the total energy demand is limited and comparable to the acidic “hot-water” methods, resulting in a valuable alternative.

## 4. Conclusions

This work compares different “green” routes for the extraction of pectin from food waste, such as orange peels recovered from household.

The comparison was based on different parameters: yield, esterification degree (which is important in view of fabricating hydrogels), and energy investment. The experimental results showed that samples of pectin obtained through different extraction methods have different morphologies, thermogravimetric properties and esterification degrees. Our analysis reveals that the rapid solid–liquid dynamic extraction (RSLD) is the method that enables the processing of a higher amount of orange peel, but it is characterized by the lowest extraction yield. Even if when we consider the energy consumption related to only laboratory instrumentations RSLD consumes the lowest amount of energy, this class of extractions requires the employment of huge amount of ethanol, reducing the sustainability of the whole procedure and significantly enhancing the total energy demand for producing pectin. Considering these aspects all together, the RSLD method is not recommended, unless for the study of further optimizations.

“Hot-water”-based methods are more efficient (higher extraction yields) and enable to obtain high-quality pectin. In particular, when “hot-water” extraction is assisted by citric acid, both extraction yield and DE% reach their maximum value (21 and 82.5%, respectively). Simultaneously, the consumption of ethanol is limited, and the total energy (instruments + chemicals embodied energy) required for producing pectin reaches its lower value. As a result, acidic “hot-water” extraction is the most sustainable method for obtaining HM pectin.

If LM pectin is desired, microwave-assisted extraction performed directly on fresh orange peel is a good alternative; even if it enables the obtainment of a lower extraction yield, it limits the amount of ethanol employed during pectin precipitation and the total energy demand is comparable to that of the acidic “hot-water” method.

In conclusion, this study provides a useful guideline for setting the production of pectin from food waste.

## Figures and Tables

**Table 1:** Comparison between different extraction protocols, in terms of experimental steps, extraction yield, esterification degree, thermogravimetric properties and morphology of extracted pectin.

Extraction	Peels	Method	Yield	DE	TGA Analysis	Morphology
1	fresh pieces	hot water MW pretreatment LSR = 20, T = 70 °C, t = 60 min	10%	78.9		almost flat surface
2	fresh pieces	hot water MW pretreatment LSR = 40, T = 90 °C, t = 180 min	10%	60	T_50%_ = 292 °C DTG_max_ = 228 °C mass loss@650 °C = 66%	flat surface
3	ground	hot water MW pretreatment LSR = 20, T = 70 °C, t = 60 min	3.5%	62.5		stressed surface
4	ground	acidic hot water LSR = 20, T = 70 °C, t = 60 min	21 %	82.5	T_50%_ = 304 °C DTG_max_ = 243 °C mass loss@650 °C = 74%	stressed surface
5	fresh pieces	RSLD MW pretreatment LSR = 4, t = 300 min	1.4%	82.3		
6	ground	RSLD LSR = 10, t = 180 min	1%	43.8		
7	ground	RSLD LSR = 10, t = 300 min	1%	40	T_50%_ = 332 °C DTG_max_ = 337 °C mass loss@650 °C = 74%	deep cavities and stressed surface
8	freeze-dried	acidic RSLD MW pretreatment LSR = 10, t = 300 min	1%	32.4		
9	freeze-dried	microwave LSR = 6, T = 110 °C, t = 5 min, P = 300 W	2.5%	66.7	T_50%_ = 314°C DTG_max_ = 227°C mass loss@650 °C = 71%	almost flat and regular surface
10	ground	microwave LSR = 6, T = 110 °C, t = 5 min, P = 300 W	6.2%	43.8		
commercial			-	50	T_50%_ = 266 °C DTG_max_ =231 °C mass loss@650 °C= 76%	granular shape

## Data Availability

The data presented in this study are available on request from the corresponding author.

## Supplementary Materials

The following are available online. Tables S1–S12 contain details on the calculation of estimated energy demand for the different extraction protocols. Figure S1: Morphological characterization of pectin extracted through variations of “hot-water” extractions. Figure S2: FT-IR characterization of pectin derived from “hot-water” extraction (extraction 3) and depectinated residues.

## Sample Availability

Samples of the compounds are available from the authors.
