# Supplementary material for: Assessing Green Methods for Pectin Extraction from Waste Orange Peels
Source: Molecules. 2021 Mar 21;26(6):1766. doi: 10.3390/molecules26061766 (PMC8004147; doi:10.3390/molecules26061766)
Supplement: Supplementary file 1 [file molecules-26-01766-s001.pdf]

molecules

Article

Assessing Green Methods for Pectin Extraction from Waste Orange Peels

Laura Benassi [1,2], Ivano Alessandri [2,3,4,5,*] and Irene Vassalini [2,3,4,5,*]

1 B+LabNet, Environmental Sustainability Laboratory, University of Brescia, via Branze 45, 25123 Brescia, Italy; laura.benassi@unibs.it
2 INSTM, U. d. R. Brescia, via Branze 38, 25123 Brescia, Italy
3 Chemistry for Technologies Laboratory, Mechanical and Industrial Engineering Department, University of Brescia, via Branze 38, 25123 Brescia, Italy
4 CNR-INO, via Branze 38, 25123 Brescia, Italy
5 Department of Information Engineering, University of Brescia, via Branze 38, 25123 Brescia, Italy
* Correspondence: ivano.alessandri@unibs.it (I.A.); irene.vassalini@unibs.it (I.V.); Tel.: +39-030-3715475 (I.A.); +39-030-3715405 (I.V.)

Tables S1–S12 contain details on the calculation of estimated energy demand for the different extraction protocols.

Figure S1: Morphological characterization of pectin extracted through variations of "hot-water" extractions.

Figure S2: Fourier Transform Infrared (FT-IR) characterization of pectin derived from "hot-water" extraction (extraction 3) and depectinated residues.

Table S1. Calculation of the energy consumed by each instrument.

experimental step	Power (W)	Time (s)	Energy (kJ)
microwave (pretreatment)	550	300	165
blender (grinding)	1500	6	9
RSLD 3h	100	10,800	1080
RSLD 5h	100	18,000	1800
centrifuge	100	1860	186
microwave (extraction)	300	600	180
freeze-drying	3520	36,000	126,720
hot plate 1h	39	60	2340
hot plate 3h	39	180	7020

Table S2. Calculation of the energy consumed by instruments for the production of 10g of pectin, following the protocol of extraction 1. Due to the low extraction yield (10%), some of the single operations had to be repeated 7 times.

Extraction 1.

	Operation	Energy Single Rep (kJ)	n° Rep	Tot Energy (MJ)
microwave (pretreatment)	yes	165	7	1.155
blender (grinding)	no	0	0	0
RSLD 3h	no	0	0	0
RSLD 5h	no	0	0	0
centrifuge	yes	186	7	1.302
microwave (extraction)	no	0	0	0
freeze-drying	no	0	0	0
hot plate 1h	yes	2340	7	16.38

**hot plate 3h**	no	0	0	0
	**tot energy**	2691		18.837

Table S3. Calculation of the energy consumed by instruments for the production of 10g of pectin, following the protocol of extraction 2. Due to the low extraction yield (10%), some of the single operations had to be repeated 7 times.

Extraction 2.

	Operation	Energy Single Rep (kJ)	n° Rep	Tot Energy (MJ)
**microwave (pretreatment)**	yes	165	7	1.155
**blender (grinding)**	no	0	0	0
**RSLD 3h**	no	0	0	0
**RSLD 5h**	no	0	0	0
**centrifuge**	yes	186	7	1.302
**microwave (extraction)**	no	0	0	0
**freeze-drying**	no	0	0	0
**hot plate 1h**	no	0	0	0
**hot plate 3h**	yes	7020	7	49.14
	**tot energy**	7371		51.597

Table S4. Calculation of the energy consumed by instruments for the production of 10g of pectin, following the protocol of extraction 3. Due to the low extraction yield (3.5%), some of the single operations had to be repeated 15 times.

Extraction 3.

	Operation	Energy Single Rep (KJ)	n° Rep	Tot Energy (MJ)
**microwave (pretreatment)**	yes	165	15	2.475
**blender (grinding)**	yes	9	15	0.135
**RSLD 3h**	no	0	0	0
**RSLD 5h**	no	0	0	0
**centrifuge**	yes	186	15	2.79
**microwave (extraction)**	no	0	0	0
**freeze-drying**	no	0	0	0
**hot plate 1h**	yes	2340	15	35.1
**hot plate 3h**	no	0	0	0
	**tot energy**	2700		40.500

Table S5. Calculation of the energy consumed by instruments for the production of 10g of pectin, following the protocol of extraction 4. Due to the low extraction yield (21%), some of the single operations had to be repeated 4 times.

Extraction 4.

	Operation	Energy Single Rep (kJ)	n° Rep	Tot Energy (MJ)
**microwave (pretreatment)**	yes	165	4	0.66
**blender (grinding)**	yes	9	4	0.036
**RSLD 3h**	no	0	0	0
**RSLD 5h**	no	0	0	0
**centrifuge**	yes	186	4	0.744

**microwave (extraction)**	no	0	0	0
**freeze-drying**	no	0	0	0
**hot plate 1h**	yes	2340	4	9.36
**hot plate 3h**	no	0	0	0
	**tot energy**	2700		10.8

Table S6. Calculation of the energy consumed by instruments for the production of 10g of pectin, following the protocol of extraction 5. Due to the low extraction yield (1.4%), some of the single operations had to be repeated 3 times.

Extraction 5.

	Operation	Energy Single Rep (kJ)	n° Rep	Tot Energy (MJ)
**microwave (pretreatment)**	yes	165	3	0.495
**blender (grinding)**	no	0	0	0
**RSLD 3h**	no	0	0	0
**RSLD 5h**	yes	1800	3	5.4
**centrifuge**	yes	186	3	0.558
**microwave (extraction)**	no	0	0	0
**freeze-drying**	no	0	0	0
**hot plate 1h**	no	0	0	0
**hot plate 3h**	no	0	0	0
	**tot energy**	2151		6.453

Table S7. Calculation of the energy consumed by instruments for the production of 10g of pectin, following the protocol of extraction 6. Due to the low extraction yield (1%), some of the single operations had to be repeated 9 times.

Extraction 6.

	Operation	Energy Single Rep (kJ)	n° Rep	Tot Energy (MJ)
**microwave (pretreatment)**	no	0	0	0
**blender (grinding)**	yes	9	9	0.081
**RSLD 3h**	yes	1080	9	9.72
**RSLD 5h**	no	0	0	0
**centrifuge**	yes	186	9	1.674
**microwave (extraction)**	no	0	0	0
**freeze-drying**	no	0	0	0
**hot plate 1h**	no	0	0	0
**hot plate 3h**	no	0	0	0
	**tot energy**	1750		11.475

Table S8. Calculation of the energy consumed by instruments for the production of 10g of pectin, following the protocol of extraction 7. Due to the low extraction yield (1%), some of the single operations had to be repeated 9 times.

Extraction 7.

	Operation	Energy Single Rep (kJ)	n° Rep	Tot Energy (MJ)
**microwave (pretreatment)**	no	0	0	0
**blender (grinding)**	yes	9	9	0.081
**RSLD 3h**	no	0	0	0
**RSLD 5h**	yes	1800	9	16.2
**centrifuge**	yes	186	9	1.674
**microwave (extraction)**	no	0	0	0
**freeze-drying**	no	0	0	0
**hot plate 1h**	no	0	0	0
**hot plate 3h**	no	0	0	0
	**tot energy**	1995		17.995

Table S9. Calculation of the energy consumed by instruments for the production of 10g of pectin, following the protocol of extraction 8. Due to the low extraction yield (1%), some of the single operations has to be repeated 9 times. On the contrary, we decided to consider freeze-drying just once, since the chamber of the freeze-dryer was big enough to host the amount of orange peel (OP) necessary to produce 10 g of pectin.

Extraction 8.

	Operation	Energy Single Rep (kJ)	n° Rep	Tot Energy (MJ)
**microwave (pretreatment)**	yes	165	9	1.485
**blender (grinding)**	no	0	0	0
**RSLD 3h**	no	0	0	0
**RSLD 5h**	yes	1800	9	16.2
**centrifuge**	yes	186	9	1.674
**microwave (extraction)**	no	0	0	0
**freeze-drying**	yes	126,720	1	126.72
**hot plate 1h**	no	0	0	0
**hot plate 3h**	no	0	0	0
	**tot energy**	128,871		146.079

Table S10. Calculation of the energy consumed by instruments for the production of 10g of pectin, following the protocol of extraction 9. Due to the low extraction yield (2.5%), some of the single operations had to be repeated more times. In particular, the sample holder of the microwave was very small and could only host 2g of OP, so we decided to group 6 samples before performing centrifugation. In addition, we decided to consider freeze-drying just once, since the chamber of the freeze-dryer was big enough to host the amount of OP necessary to produce 10 g of pectin.

Extraction 9.

	Operation	Energy Single Rep (kJ)	n° Rep	Tot Energy (MJ)
**microwave (pretreatment)**	no	0	0	0
**blender (grinding)**	no	0	0	0

**RSLD 3h**	no	0	0	0
**RSLD 5h**	no	0	0	0
**centrifuge**	yes	186	33	6.138
**microwave (extraction)**	yes	180	198	35.64
**freeze-drying**	yes	126,720	1	126.72
**hot plate 1h**	no	0	0	0
**hot plate 3h**	no	0	0	0
	**tot energy**	127,086		168.498

Table S11. Calculation of the energy consumed by instruments for the production of 10g of pectin, following the protocol of extraction 11. Due to the low extraction yield (6.2%), some of the single operations had to be repeated more times. In particular, the sample holder of the microwave was very small and could only host 2g of OP, so we decided to group 6 samples before performing centrifugation.

Extraction 10.

	**Operation**	**Energy Single Rep (kJ)**	**n° Rep**	**Tot Energy (MJ)**
**microwave (pretreatment)**	no	0	0	0
**blender (grinding)**	yes	9	13	0.117
**RSLD 3h**	no	0	0	0
**RSLD 5h**	no	0	0	0
**centrifuge**	yes	186	13	2.418
**microwave (extraction)**	yes	180	78	14.04
**freeze-drying**	no	0	0	0
**hot plate 1h**	no	0	0	0
**hot plate 3h**	no	0	0	0
	**tot energy**	375		16.575

Table S12. Calculation of the embodied energy of the chemicals involved in the different extraction protocols for the production of 10 g of pectin.

**Extraction**	**OP Mass (g)**	**Water Volume (ml)**	**n° Rep**	**Total Ethanol Volume (ml)**	**Citric Acid (kg)**	**Total Ethanol Embodied Energy (MJ)**	**Total Citric Acid Embodied Energy (MJ)**
**1**	15	300	7	2110	0	71.752449	
**2**	15	600	7	4210	0	143.164839	
**3**	20	400	15	6010	0	204.375459	
**4**	15	300	4	1210	0.032	41.147139	2.74112
**5**	250	1000	3	3010	0	102.357759	
**6**	110	1100	9	9910	0	336.998469	
**7**	110	1100	9	9910	0	336.998469	
**8**	110	1100	9	9910	0.27	336.998469	23.1282
**9**	12	72	33	3574	0	121.5370866	
**10**	12	72	13	1414	0	48.0843426	

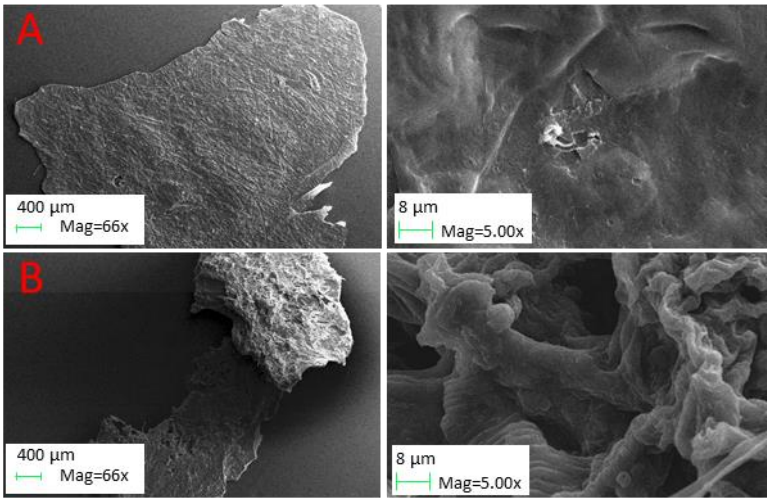

Figure S1. Low- and high-magnification SEM images for "hot-water" extracted pectin: **A**) extraction 1; **B**) extraction 3.

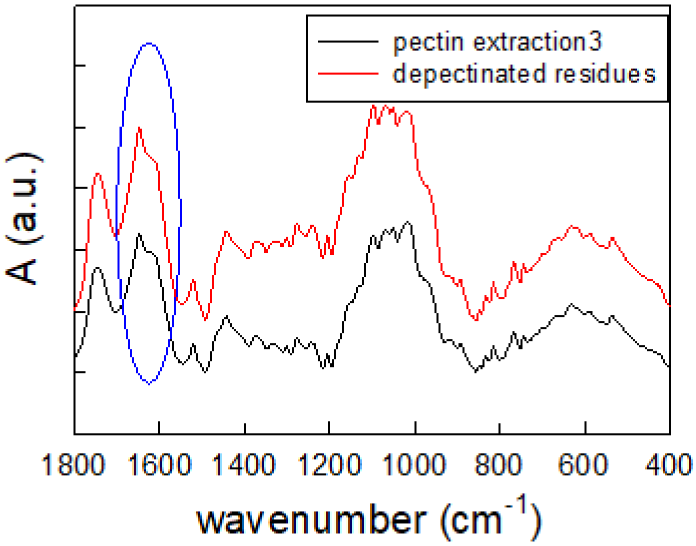

Figure S2. Comparison between the Fourier Transform Infrared (FT-IR) spectra of pectin derived from extraction 3 and de-pectinated residues. Impurities are highlighted.
